# Molecular prevalence of *Anaplasma* spp. in cattle and assessment of associated risk factors in Northeast Thailand

**DOI:** 10.14202/vetworld.2023.1702-1707

**Published:** 2023-08-24

**Authors:** Tossapol Seerintra, Bhuripit Saraphol, Tongjit Thanchomnang, Supawadee Piratae

**Affiliations:** 1 Faculty of Veterinary Sciences, Mahasarakham University, Maha Sarakham, 44000, Thailand; 2 Faculty of Medicine, Mahasarakham University, Maha Sarakham, 44000, Thailand; 3 One Health Research Unit, Mahasarakham University, Maha Sarakham, 44000, Thailand; 4 Veterinary Infectious Disease Research Unit, Mahasarakham University, Maha Sarakham, 44000, Thailand

**Keywords:** anaplasmosis, beef cattle, blood parasite, dairy cattle, molecular detection

## Abstract

**Background and Aim:**

*Anaplasma* spp. are common rickettsia species described in ruminant hosts, including cattle. The clinical signs of anaplasmosis range from asymptomatic to mortality. However, there are insufficient studies on epidemiology surveys of this blood pathogen. This study aimed to estimate the prevalence and risk factors of anaplasmosis in beef and dairy cattle in Northeast, Thailand.

**Materials and Methods:**

A total of 187 blood samples of beef and dairy cattle were collected from five provinces in Northeast Thailand. *Anaplasma* spp. infections were screened by microscopic examination and polymerase chain reaction targeting specific genes (*msp4* gene for *Anaplasma marginale* and 16S rRNA gene for *Anaplasma platys* and *Anaplasma bovis*). Moreover, the associated risk factors for the infections were evaluated.

**Results:**

Overall, blood samples from cattle revealed that 17.6% (33/187) were positive for *Anaplasma* spp. by microscopic examination and 20.8% (39/187) were positive by DNA amplification. Of these 20.8%, 17.6% were *A. marginale* and 3.2% were *A. platys*. However, *A. bovis* infection was not detected. Infection with *Anaplasma* spp. and *A. marginale* showed a significant association with breed and gender (p < 0.05) while age and packed cell volume levels showed no significant statistical relationship between *Anaplasma* spp. infected and uninfected animals.

**Conclusion:**

This study indicated that anaplasmosis is distributed in beef and dairy cattle in Thailand; therefore, prevention and control strategies for these pathogens should be improved. This information will benefit veterinarians and cowherds by avoiding vector exposure and eliminating tick breeding sites.

## Introduction

*Anaplasma* is a Gram-negative intracellular bacterium in the family Anaplasmataceae which exists in the blood cells of a variety of mammals, including cattle and people [1]. Moreover, this pathogen is also found exclusively within membrane-bound vacuoles in the invertebrate or tick host cytoplasm. Several types of *Anaplasma*: *Anaplasma marginale, Anaplasma centrale, Anaplasma bovis*, and *Anaplasma platys* can cause bovine anaplasmosis, which is a serious health problem in cattle [2–5]. The clinical signs are hemoglobinuria, anemia, fever, jaundice, loss of appetite, weight loss, decreased milk production, abortion, and even death [2]. To detect bovine anaplasmosis, microscopic examination is commonly used in combination with hematological values. Moreover, serological testing for antibody detection and polymerase chain reaction (PCR) for DNA detection have been developed to diagnose bovine anaplasmosis [4].

In Thailand, which is an agricultural state located in Southeast Asia, the livestock industry has been hampered by severe tick-borne hemoparasites. Beef and dairy cattle are predominant animals in Northeast Thailand and provide an important source of meat, horns, milk products, leather, land plowing, and transportation of people and crops [6]. Bovine anaplasmosis in Thailand has been reported continually and its consequences impact economic losses in cattle production [7]. Two species of *Anaplasma* that have been endemic in this region are *A. marginale* and *A. platys*. The previous studies by Saetiew *et al*. [8], Jirapattharasate *et al*. [9], and Nguyen *et al*. [10] reported that *A. marginale* is the most prevalent tick-borne pathogen in North, Northeastern, and Western Thailand with a prevalence rate of approximately 8%–40%. However, there is less updated information on the epidemiology of *Anaplasma* spp. infection in cattle.

This study aimed to estimate the prevalence of *Anaplasma* spp. infections in beef and dairy cattle in Northeast, Thailand by microscopic examination and PCR. Moreover, the associated risk factors for *Anaplasma* spp. infections in naturally infected cattle were also determined.

## Materials and Methods

### Ethical approval

All experimental procedures involving animals were approved by the Institutional Animal Care and Use Committee, Mahasarakham University (IACUC-MSU-26/2022).

### Study period and location

**Figure-1:**
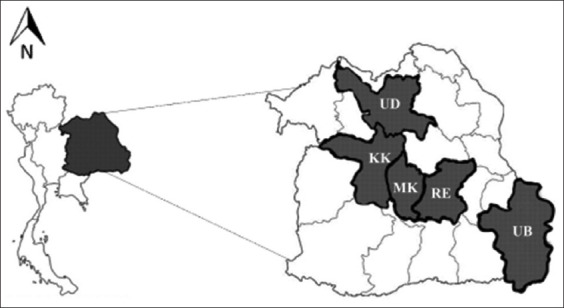
Map showing the area of cattle blood sampling in five provinces in the Northeastern Region of Thailand, consisting of Udonthani (UD), Khonkhan (KK), Mahasarakham (MK), Roi-et (RE), and Ubonrachathani (UB). [Source: https://shorturl.at/jrOST].

The cross-sectional study was conducted from June 2021 to May 2022. The blood samples were collected from cattle in smallholder farms in Khon Kaen, Maha Sarakham, Roi Et, Ubon Ratchathani, and Udon Thani provinces of Thailand (Figure-1).

### Sample collection and study area

In total, 187 blood samples (106 samples from beef cattle and 81 samples from dairy cattle) were collected (approximately 3–5 mL from each) from the jugular vein or coccygeal vein in ethylenediaminetetraacetic acid anticoagulant tubes. The information on age, breed, gender, capillary refill time, and body condition score (BCS) were also recorded. Blood samples were transported on ice to the laboratory at the Faculty of Veterinary Sciences of Mahasarakham University. All blood samples were screened for *Anaplasma* infections using thin blood smear technique and measurement of the packed cell volume (PCV) levels which were performed on the same day of blood collection. The remaining blood was stored at –20°C until DNA extraction.

### Microscopic examination using the blood smear technique

To perform this technique, approximately 10–20 μL blood was poured onto a slide and spread. The blood smear slides were then air-dried for 5–10 s, fixed with 100% methanol for 5 min, and stained with 10% Giemsa’s solution for 15 min. Blood films were observed in the monolayer fields under a light microscope (Olympus, Japan) to determine the presence of parasites.

### DNA extraction and PCR methods

DNA was extracted from whole blood (200 μL) following the GF-1 blood DNA extraction kit protocol (Vivatis, Malaysia). DNA of each sample was stored at −20°C for long-term preservation. Each extracted DNA sample was examined for blood parasitic infections by PCR or nested PCR. The first step of PCR used universal primers of the DNA belonging to Anaplasmataceae parasites for screening infections. In the next step, positive samples for *Anaplasma* infections were examined for species detection by specific primers (Table-1) [11–14]. For the PCR reaction, approximately 10–50 ng of the extracted DNA was used as a template in a 25 μL reaction containing 1 μL of each primer (10 μmol/L), 1.5 mM MgSO_4_, 0.2 mM deoxynucleotide triphosphate, 1× PCR buffer, and 1 U of *Taq* Polymerase (Fermentas, USA). The reaction conditions comprised 35 cycles of denaturation for 45 s at 95°C, annealing for 45 s at 55°C–60°C, and extension for 1.5 min at 72°C using a PCR thermocycler (Biometra, Göttingen, Germany). The PCR products were electrophoresed running in 1% agarose gel stained with ViSafe Red Gel Stain (Vivantis, Malaysia) and visualized under ultraviolet light to check for positive amplifications.

**Table-1:** Primers for PCR amplifications.

Pathogens	Primers	Target genes	Annealing temperature	Size of product (bp)	Reference
Anaplasmataceae	EHR16SD (5’ GGTACCYACAGAAGAAGTCC 3’) ESR16SR (5’ TAGCACTCATTTACAGC 3’)	16S rRNA	55	345	[11]
*A. marginale*	MSP43 (5’ GGGAGCTCCTATGAATTACAGAGAATTGTTTAC 3’) MSP45 (5’ CCGGATCCTTAGCTGAACAGGAATCTTGC 3’)	*msp4*	56	849	[12]
*Anaplasma* (outer primer)	EE1 (5’ TCCTGGCTCAGAACGAACGCTGGCGGC 3’) EE2 (5’ AGTCACTGACCCAACCTTAAATGGCTG 3’)	16S rRNA	60	1,430	[13]
*A. platys* (inner primer)	APf (5’ AAGTCGAACGGATTTTTGTC 3’) APr (5’ CTTTAACTTACCGAACC 3’)	16S rRNA	55	506	[13]
*A. bovis* (inner primer)	AB1f (5′ CTCGTAGCTTGCTATGAGAAC 3′) AB1r (5′ TCTCCCGGACTCCAGTCTG 3′)	16S rRNA	55	551	[14]

PCR=Polymerase chain reaction, *A. marginale=Anaplasma marginale, A. platys=Anaplasma platys, A. bovis=Anaplasma bovis*

### Levels of PCV examination

Levels of PCV were evaluated as the height of the pack red cell column in a microhematocrit tube after centrifugation. The PCV was measured by filling blood directly into the microhematocrit tube, then centrifuged at 15,000× *g* for 3 min. The height of the total blood column and the height of the red cell layer was measured within 1 min after centrifugation was stopped.

### Statistical analysis

The presence of blood parasites was determined and the percentages of infection were calculated. Confidence intervals (CI) were also used to compare the prevalence of parasitic infections. The association between blood parasite infections with other factors including gender, age, breed, and PCV levels was compared using Pearson’s Chi-squared test. Statistical differences were considered when p < 0.05.

## Results

Blood samples of beef and dairy cattle were collected from five provinces in the Northeastern region of Thailand which are Khon Kaen (n = 48), Maha Sarakham (n = 94), Roi Et (n = 12), Ubon Ratchathani (n = 13), and Udon Thani (n = 20). Blood samples of 187 cattle comprised 56.7% (106) of beef cattle and 43.3% (81) of dairy cattle. About 106 samples of beef cattle were male 13.2% and female 86.8%. The 81 samples collected from dairy cattle were male 16% and female 84%. Animal samples were in the age range from 2 months to 10 years old (21.9% were <1 year; 66.3% were 1–6 years old; and 11.8% were >6 years old) (Table-2). All sampling animals showed capillary refill time <2 s. Animals with BCS <3 and ≥3 were 114 and 64, respectively.

**Table-2:** Characteristics of cattle and risk factors analysis.

Characteristics	No. of cattle	No. of positive with				

		*Anaplasma* spp. infected (%)	p-value	*A.* *marginale* infected (%)	p-value	*A.* *platys* infected (%)
Breed			0.03*		0.00*	
Beef cattle	106	16 (15.1)		10 (9.4)		6 (5.7)
Dairy cattle	81	23 (28.4)		23 (28.4)		0 (0)
Gender			0.00*		0.004*	
Male	27	12 (44.4)		10 (37)		2 (7.4)
Female	160	27 (16.9)		23 (14.4)		4 (2.5)
Age (years)			0.51		0.42	
Calf (0–1)	39	11 (28.2)		10 (25.6)		1 (2.6)
Adult (1–6)	118	23 (19.5)		20 (16.9)		3 (2.5)
Old (>6)	21	5 (23.8)		3 (14.3)		2 (9.5)
% PCV			0.90		0.78	
Anemia (<24)	30	6 (20)		6 (20)		0 (0)
Non anemia (≥24)	157	33 (21)		27 (17.2)		6 (3.8)

PCV=Packed cell volume, *A. marginale=Anaplasma marginale, A. platys=Anaplasma platys, A. bovis=Anaplasma bovis*

**Figure-2:**
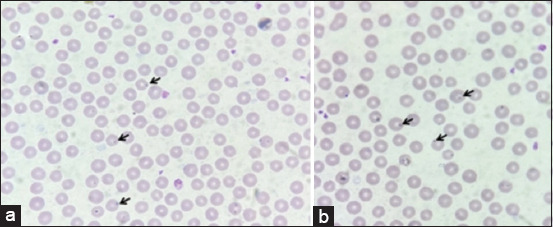
*Anaplasma marginale* infections in erythrocytes of (a) beef cattle and (b) dairy cattle.

**Figure-3:**
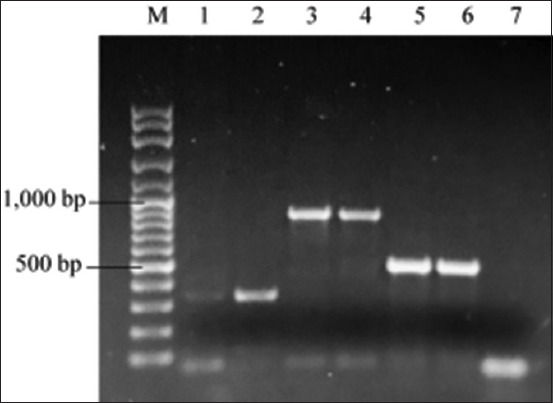
Agarose gel electrophoresis of polymerase chain reaction products. Lane M: 100 bp DNA ladder marker; Lanes 1–2: Positive samples for Anaplasmataceae at 345 bp; Lanes 3–4: Positive samples for *Anaplasma marginale* at 849 bp; Lanes 5–6: Positive samples for *Anaplasma platys* at 506 bp; Lane 7: Negative control.

Under the light microscope, *Anaplasma* spp. infections were detected in erythrocytes (Figure-2). For microscope examination, the prevalence of *Anaplasma* spp. was 17.6 %. The occurrence of Anaplasmataceae was examined by PCR based on the 16S rRNA gene. Fragments of *msp4* of *A. marginale* and 16S rRNA of *A. platys* were amplified to examine infections (Figure-3). For PCR, the overall prevalence of anaplasmosis in cattle in Northeast Thailand was 20.8% (95% CI: 15.3–27.4) based on 16S rRNA Anaplasmataceae primers. For specific primer detection, 5.3% and 3.2% of beef cattle were infected with *A. marginale* and *A. platys*. In addition, the molecular prevalence of *A. marginale* in dairy cattle was 12.3%, while no infection with *A. platys* was observed in this population. In addition, *A. bovis* infection was not discovered in this study.

Regarding breed, dairy cattle were more susceptible to *Anaplasma* infection (28.4%) than beef cattle (15.1%). Male cattle (44.4%) were more likely to be *Anaplasma* infected than females (16.9%). For PCV values, the average PCV levels in both infected and uninfected groups were in the normal range (28.5% vs. 30%). Although the infected group had a lower trend of PCV, the results showed no statistical difference between infected and uninfected groups. Moreover, there were no clinical signs in any cattle infected with *Anaplasma* spp. In addition, statistical tests of the association between *Anaplasma* infections and other factors showed infection with *Anaplasma* spp. and *A. marginale* had an association with breed and gender (p < 0.05) while age and PCV levels showed no significant statistical relationship between *Anaplasma* spp. infected and uninfected groups.

## Discussion

Anaplasmosis in cattle is a worldwide veterinary health problem, especially in tropical and subtropical regions. In this study, we screened *Anaplasma* spp. infection in beef and dairy cattle using both microscopic and molecular techniques. From this study, the overall prevalence of *Anaplasma* spp. in cattle was 20.8% based on PCR and 17.6% based on microscopic results. Although microscopic examination by direct blood smear technique is common, it is suitable for the detection of anaplasmosis during the acute phase of infection and requires an expert examiner. Polymerase chain reaction is an advantageous assay over microscopic examination because it has high sensitivity and specificity and is widely used to detect all phases of anaplasmosis infection in animals. The results indicated that the PCR method exhibited much higher sensitivity for the diagnosis of this blood parasite than the microscopic method, which is the routine method in the laboratory.

In Thailand, *Anaplasma* spp. infection in large ruminants is endemic with a higher infection rate reported in water buffalo (41%) [10] and beef cattle in the Western region (39.1%) [9]. However, the prevalence in this study is higher than the previous studies by Junsiri *et al*. [15] in cattle in the northern and northeastern regions of Thailand in 2020 (10.30%) and water buffaloes in Northeast Thailand (8%) [8]. The difference in the prevalence of anaplasmosis in cattle in Thailand could be explained by the climatic condition in each region which influences the spread of tick vectors [16], farm management, herd size, sampling period, sample size, antibiotic prevention [17], and diagnosis protocols [18]. In addition, this study notices that good management practices on the farm have been observed to be the key factor in the infection rate. In other counties, the prevalence of anaplasmosis in cattle varies from 8.7% in Mongolia [19], 9% and 17% in Punjab (Pakistan) [20], 11.1% in Pakistan [21], 15.7% in India [22], 38.53% in Ohio [23], 49.1 % in Nigeria [24], and 68.3% in Egypt [25]. The prevalence of bovine anaplasmosis in this study is reliable in range with previous epidemiological studies.

Apart from risk factor analysis, we found that the risk factors for *Anaplasma* spp. and *A. marginale* infections were significantly associated with breed and gender. Previous data also supported our finding that breed and gender had significant associations with *Anaplasma* spp. [26]. For gender, the results showed that male cattle had a higher infection rate than female cattle according to the finding in cattle in China [27] and buffalo in Pakistan [28]. For breed, the results revealed that dairy cattle are more susceptible to anaplasmosis than beef cattle. Although a previous study reported the age of the animals (below 1 year of age) showed a significant association with *Anaplasma* spp. infections [26], we found adverse results that age showed no significant relationship with infections. However, similar results in this study were also reported in water buffaloes from eight provinces of Thailand [10]. In addition, the principal clinical sign of bovine anaplasmosis was considered anemia which can be directly measured by PCV levels; however, we found PCV levels showed no significant relationship with infections according to the report of PCV levels in infected cattle in Nigeria [24]. This phenomenon may support the evidence that most cattle, especially animals that adapt well to a tropical climate show milder symptoms on infection.

## Conclusion

From this study, *Anaplasma* infections in cattle in Thailand are common and several risk factors affect the rate of *Anaplasma* spp. infection, including host (age, gender, and breed) and environments (ecosystem, farm management, herd size, etc.). In addition, *Anaplasma* spp. is transmitted by ticks, the presence of this pathogen suggests that probable vectors may occur in ecological surroundings and requires further investigation.

## Authors’ Contributions

TS: Conceptualization, sample collection, data curation, formal analysis, investigation, and methodology. BS: Sample collection and data curation. TT: Conceptualization and review and editing of the manuscript. SP: Conceptualization, methodology, and writing, review, and editing of the manuscript. All authors have read, reviewed, and approved the final manuscript.
